# Genome-Wide Characterization of *CaM*/*CML* Gene Family in Cabbage (*Brassica oleracea* var. *capitata*): Expression Profiling and Functional Implications During *Hyaloperonospora parasitica* Infection

**DOI:** 10.3390/ijms26073208

**Published:** 2025-03-30

**Authors:** Yuankang Wu, Bin Zhang, Xuehui Yao, Limei Yang, Mu Zhuang, Honghao Lv, Yong Wang, Jialei Ji, Xilin Hou, Yangyong Zhang

**Affiliations:** 1 State Key Laboratory of Vegetable Biobreeding, Institute of Vegetables and Flowers, Chinese Academy of Agricultural Sciences, Beijing 100081, China; wuyk1129@163.com (Y.W.); yaoxuehui@caas.cn (X.Y.); yanglimei@caas.cn (L.Y.); zhuangmu@caas.cn (M.Z.); lvhonghao@caas.cn (H.L.); wangyong@caas.cn (Y.W.); jijialei@caas.cn (J.J.); 2 National Key Laboratory of Crop Genetics & Germplasm Innovation and Utilization, Key Laboratory of Biology and Genetic Improvement of Horticultural Crops (East China), Ministry of Agriculture and Rural Affairs of China, Engineering Research Center of Germplasm Enhancement and Utilization of Horticultural Crops, Ministry of Education of China, Nanjing Agricultural University, Nanjing 210095, China; 3 State Key Laboratory of Vegetable Biobreeding, Beijing Vegetable Research Center, Beijing Academy of Agriculture and Forestry Science, Beijing 100097, China; brzhangbin@nercv.org

**Keywords:** downy mildew, calmodulin gene family, RNA-seq, *Brassica oleracea*, expression analysis

## Abstract

Calmodulin (CaM) and calmodulin-like proteins (CMLs) are crucial for calcium signal transduction in plants. Although *CaM*/*CML* genes have been extensively studied in various plant species, research on these genes in *Brassica oleracea* is still limited. In this study, 14 *BoCaM* and 75 *BoCML* genes were identified in the *B. oleracea* genome through a genome-wide search. Phylogenetic analysis categorized these genes, along with their homologs in *Arabidopsis* and rice, into six distinct groups. All *BoCaM*/*BoCML* genes were unevenly distributed across the nine chromosomes of *B. oleracea*, with 52 of them lacking introns. Collinearity analysis revealed that *CaM*/*CML* genes in *Arabidopsis* are present in multiple copies in the *B. oleracea* genome. Moreover, the majority of *BoCaM*/*BoCML* genes exhibited distinct expression patterns across the different tissues, indicating their role in the growth and development of *B. oleracea*. A clustering heatmap of *BoCaM*/*BoCML* gene expression showed distinct patterns before and four days after *Hyaloperonospora parasitica* infection, dividing the genes into five groups based on their expression patterns. Notably, *BoCML46-2* is significantly downregulated in both susceptible and resistant materials, suggesting that it plays an important role in responding to *H. parasitica* infection. This study conducted a comprehensive survey of the *BoCaM/BoCML* gene family in *B. oleracea*. It could serve as a theoretical foundation for further functional identification and utilization of family members and their role in the interaction between *B. oleracea* and *H. parasitica*.

## 1. Introduction

Plants encounter numerous biotic and abiotic stresses during their growth and development, which can lead to significant reductions in yield. They respond to these pressures through intricate internal signaling mechanisms [1]. Serving as a ubiquitous intracellular coordinator, calcium (Ca^2+^) mediates decoding of environmental stimuli into stress-adaptive signaling cascades. Almost all types of environmental stresses lead to changes in the cytosolic free Ca^2+^ levels within plant cells and affect the movement of Ca^2+^ between cellular organelles [2,3]. When faced with environmental stresses, plants translate Ca^2+^ signals into specific downstream responses through a complex array of calcium sensor proteins, thereby precisely regulating internal homeostasis [4]. In *Arabidopsis*, over 250 calcium sensor proteins have been identified, including calcineurin B-like proteins (CBLs), calmodulin (CaM), calmodulin-like proteins (CMLs), calcium-dependent protein kinases (CPKs), and calcium and calmodulin-dependent protein kinase (CCaMK), and all of them contain different numbers of EF-hand motifs [5,6,7,8,9,10].

CaMs and CMLs are essential types of Ca^2+^ sensors and are crucial components in Ca^2+^ signal transduction [11]. *CaMs*, which contain four EF-hand motifs, are conserved Ca^2+^ sensors found in both plants and animals [12]. On the other hand, *CMLs*, which typically contain 1–6 EF-hand motifs, show some sequence similarity to *CaM* and display structural variations in plants [13]. Genome-wide identification and analysis of *CaM/CML* genes have been conducted for numerous plant species, including *Arabidopsis* (7 *CaMs* and 50 *CMLs*), rice (*Oryza sativa*, 5 *CaMs* and 32 *CMLs*), and *Brassica napus* (25 *CaMs* and 168 *CMLs*) [7,14,15]. Although *CMLs* and *CaMs* are homologous, the number of *CMLs* in plants is significantly higher than that of *CaMs*. The roles of *CaMs* and *CMLs* in responding to stress have been well documented. In *Arabidopsis*, the *AtCaM3* knockout mutant exhibits reduced heat tolerance, whereas transgenic lines overexpressing *AtCaM3* demonstrate enhanced heat tolerance [16]. *AtCML8* and *AtCML9* enhance *Arabidopsis* resistance to *Pseudomonas syringae* via the ABA and SA pathways, respectively [17]. The *AtCML24* gene plays a role in inhibiting pathogen-induced nitric oxide (NO) generation [18]. In cotton, GhCML11 interacts with GhMYB108, acting as a positive regulator in defense against *Verticillium dahliae* infection [19]. In pepper, *CaCML13* acts as a positive regulator of immunity against *Ralstonia solanacearum* inoculation by forming a positive feedback loop with CabZIP63 [20]. Furthermore, *OsCaML2* can serve as a target gene for osa-miR1432, and its overexpression enhances resistance to *Xanthomonas oryzae* pv. *Oryzae* [21]. Recently, *SlCML55* has been identified as a novel calmodulin-like protein in tomato that negatively regulates plant immunity by inhibiting *Phytophthora capsici* infection and interacting with the SA signaling pathway [22]. Although the roles of *CaMs*/*CMLs* in response to various stimuli have been extensively studied in several plant species, a comprehensive genome-wide analysis of the *CaM*/*CML* gene families in cabbage (*Brassica oleracea* var. *capitata* L.) is lacking, and the potential functions of cabbage *CaM*/*CML* genes remain unclear.

Cabbage is one of the most widely cultivated vegetables globally, with a significant quantity of this crop consumed annually. The production of cabbage is significantly impacted by four prominent diseases: wilt, black rot, root knot disease, and downy mildew. Downy mildew, caused by the oomycete *H. parasitica*, has emerged as a significant threat to cabbage production in recent years [23]. However, research efforts directed towards identifying resistance genes against downy mildew in cabbage remain scarce. Investigating cabbage resistance genes through various approaches is essential for elucidating the molecular mechanisms of downy mildew resistance and accelerating molecular breeding for resistance.

In this study, 14 *BoCaM* and 75 *BoCML* genes were identified in *B. oleracea* based on their homology to those in *Arabidopsis thaliana.* The characteristics of the *B. oleracea* CaM/CML protein including molecular weight, theoretical pI, grand average of hydropathicity, and subcellular localization. Additionally, intron-exon structures, chromosomal localizations, EF-hand motifs, and phylogenetic relationships of the *BoCaM* and *BoCML* genes were analyzed. Additionally, the tissue-specific expression of *BoCaM* and *BoCML* genes in various cabbage tissues was analyzed, and their differential expression profiles in response to *Hyaloperonospora parasitica* infection were compared between resistant and susceptible cabbage varieties. These findings lay a theoretical foundation for future studies on the functional identification and utilization of *BoCaM*/*BoCML* genes, particularly in understanding their role in the interaction between *B. oleracea* and downy mildew.

## 2. Results

### 2.1. Identification and Characterization of BoCaM/BoCML Genes in B. oleracea

To identify the putative *CaM* and *CML* genes in *B. oleracea*, 7 *CaM* and 50 *CML* genes of *Arabidopsis* were used to blastp against *B. oleracea* genome protein sequence in the BRAD database (Supplementary Table S1). The results of this blastp search were then analyzed using Pfam, InterProScan, and the NCBI CD-search tool to identify the *CaM/CML* genes in *B. oleracea*. A total of 14 *BoCaM* and 75 *BoCML* genes have been identified and were named as *BoCaM1-1* to *BoCML50-2* based on their homologous relationship to *Arabidopsis CaM/CML* genes. The lengths of the *BoCaM*/*BoCML* genes ranged from 78 amino acids (*BoCML28-2*) to 436 amino acids (*BoCML21-2*), with varying numbers of EF-hand motifs. Their isoelectric points (pI) and theoretical molecular weights (MW) ranged from 4.00 to 6.89 and 8.55 to 49.74 kDa, respectively. Notably, the Grand Average of Hydropathicity (GRAVY) results indicate that most *BoCaM*/*BoCML* genes exhibit relatively high hydrophilicity, while *BoCML15-1* and *BoCML47* display relatively high hydrophobicity. Subcellular localization predictions revealed that the majority (62) of *BoCaM*/*BoCML* genes are located in the nucleus, while only *BoCML21-2* is located in the mitochondrion. Furthermore, the instability index and aliphatic index of *BoCaMs/BoCMLs* were also provided (Supplementary Table S2).

### 2.2. Phylogenetic Analysis, Protein Motifs, and Gene Structure of BoCaM/BoCML Genes

**Figure 1:**
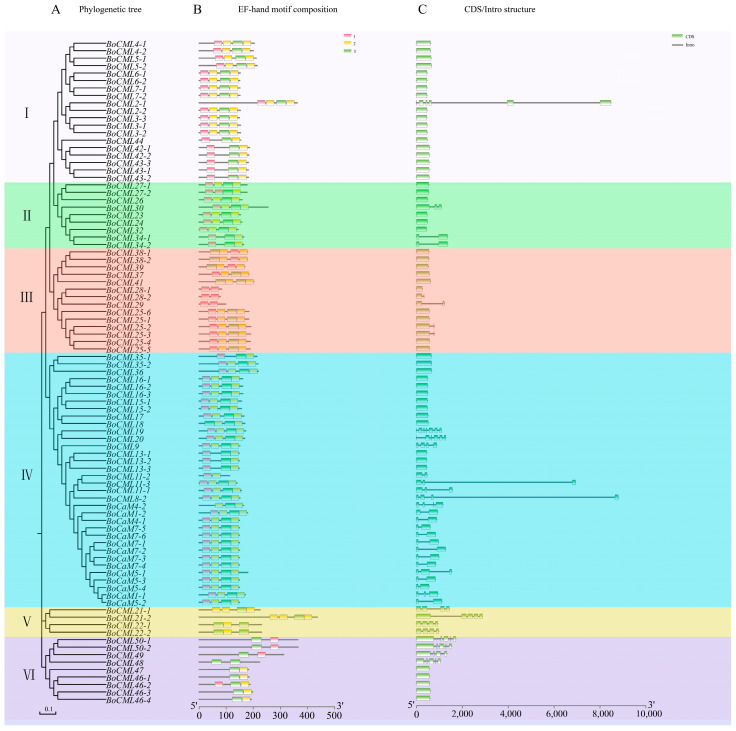
Characterization of the identified *BoCaM*/*BoCML* genes in *B. oleracea*. (**A**) Phylogenetic relationships and classification of BoCaM/BoCML proteins. (**B**) Distribution of conserved EF-hand motifs among the BoCaM/BoCML proteins. (**C**) Exon–intron structure of *BoCaM*/*BoCML* genes.

A total of 14 *BoCaM* and 75 *BoCML* genes were classified into six groups (Groups I–VI) based on their evolutionary relationships (Figure 1). Notably, Group IV has the largest number of members, including all *BoCaM* genes, whereas Group V has only four members (*BoCML21-1*, *BoCML21-2*, *BoCML22-1*, *BoCML22-2*). *BoCML* genes originating from the same *Arabidopsis* gene (e.g., *BoCML25-1* to *BoCML25-6*, *BoCML46-1* to *BoCML46-4*) are clustered together in the same group. Additionally, there are 63 members with four EF-hand motifs, 14 members with three EF-hand motifs, and 12 members with two EF-hand motifs. All *BoCaM* genes, except for *BoCaM4-2,* which possesses three EF-hand motifs, exhibit four EF-hand motifs, which corresponds to their relatively distant placement from other *BoCaM* genes in the phylogenetic tree. Furthermore, the majority (52) of *CaM/CML* members have only a single exon without intron regions, and their genes are relatively short. However, *BoCML2-1*, *BoCML11-3*, and *BoCML8-2* have large introns, resulting in gene lengths of approximately 8000 bp. Overall, genes within the same subcategories exhibit close evolutionary relationships, considerable sequence similarity, and comparable genetic architectures.

### 2.3. Phylogenetic Relationship of CaM/CML Genes Among B. oleracea, Arabidopsis, and Rice

**Figure 2:**
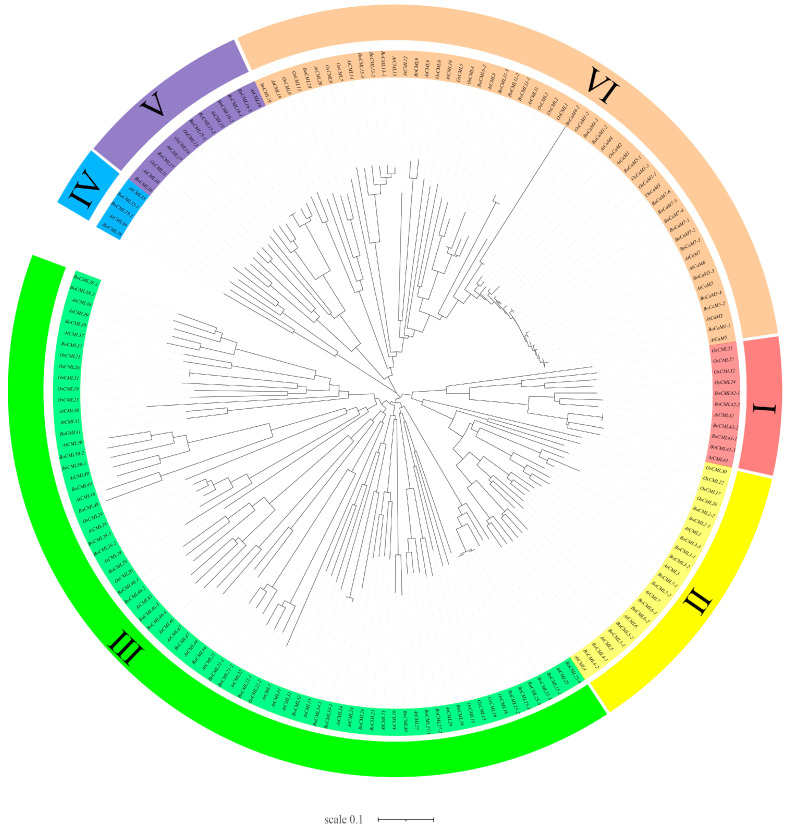
Phylogenetic tree of *CaM*/*CML* proteins in *B. oleracea*, *Arabidopsis*, and rice constructed using the neighbor-joining method.

Protein sequences for 14 *BoCaM* and 75 *BoCML* genes in *B. oleracea*, 7 *AtCaM* and 50 *AtCML* genes in *Arabidopsis*, and 5 *OsCaM* and 32 *OsCML* genes in *Oryza sativa* were obtained. Phylogenetic analysis was performed using the full protein sequences of these genes from *B. oleracea*, *Arabidopsis*, and rice (Figure 2). All *CaM*/*CML* genes were clustered into six groups (Groups I–VI). The results suggest that most *CaM*/*CML* genes originate from a common ancestor. Group III contains the largest number of members (75), whereas Group IV has the fewest members (*BoCML36*, *AtCML36*, *AtCML35*, *BoCML35-1*, *BoCML35-2*), with no *OsCaM*/*OsCML* genes present. In the clustering analysis, all *CaM* genes, whether from dicotyledonous (*B. oleracea*, *Arabidopsis*) or monocotyledonous (rice) plants, were grouped into Cluster VI. Additionally, most of the *BoCaM*/*BoCML* genes are homologous to those in *Arabidopsis* and rice. These findings suggest that *CaM*/*CML* genes are conserved across diverse plant species.

### 2.4. Chromosome Distribution and Collinearity Analysis of BoCaM/BoCML Genes

**Figure 3:**
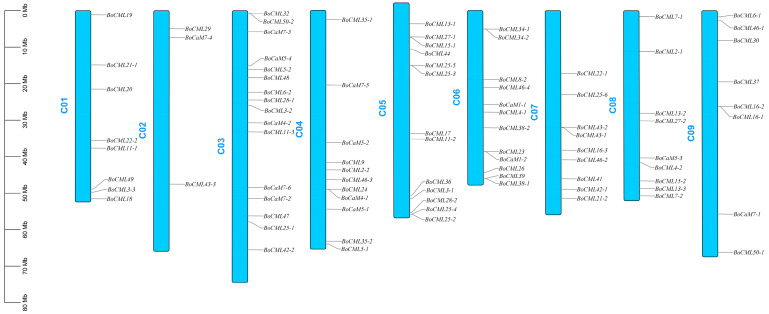
Chromosome distribution of *BoCaM*/*BoCML* genes in *B. oleracea*.

To analyze the chromosomal localization of *BoCaM* and *BoCML* genes, the genes were mapped onto the chromosomes of the *B. oleracea* genome (*B. oleracea* JZS V2.0) (Figure 3). All 89 *BoCaM*/*BoCML* genes were located on the nine chromosomes of *B. oleracea*. The largest number (16) of *BoCaM*/*BoCML* genes were located on chromosome C03, while only 3 *BoCaM*/*BoCML* genes (*BoCML29*, *BoCaM7-4*, and *BoCML43-3*) were mapped to chromosome C02. The remaining *BoCaM/BoCML* genes were unevenly distributed across the other chromosomes.

**Figure 4:**
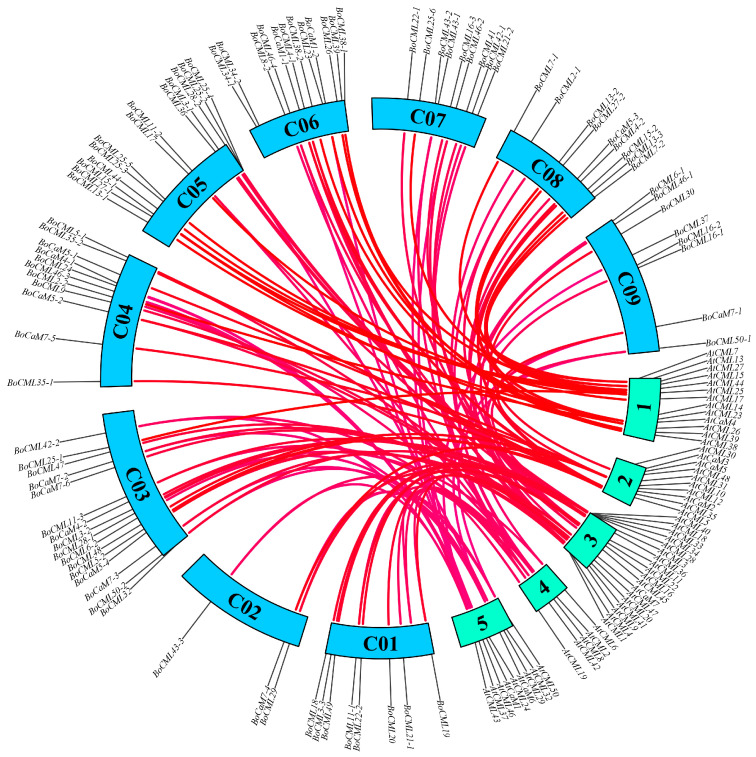
Collinearity analysis of *CaM*/*CML* genes in *Arabidopsis* and *B. oleracea*.

To gain deeper insights into the evolutionary lineage of *CaM* and *CML* genes in *B. oleracea* and *Arabidopsis thaliana*, the duplication events of candidate *CaM*/*CML* genes within their genomes were investigated (Figure 4). Among the *CML* genes in *Arabidopsis*, only 2 (*AtCML21* and *AtCML49*) lacked homologs in *B. oleracea*, whereas the remaining 55 *CaM*/*CML* genes in *Arabidopsis* had at least one homolog in *B. oleracea*. In the genomes of *B. oleracea* and *Arabidopsis*, a total of 959 collinear blocks were identified, encompassing 50,429 genes. This set included 89 *BoCaM*/*BoCML* genes from *B. oleracea* and 57 *AtCaM*/*AtCML* genes from *A. thaliana*. For instance, *AtCaM7* had six homologous genes within the genome of *B. oleracea*, whereas *AtCML9* had only a single homolog.

### 2.5. Expression Profiling of BoCaM/BoCML Genes in Different Tissues

**Figure 5:**
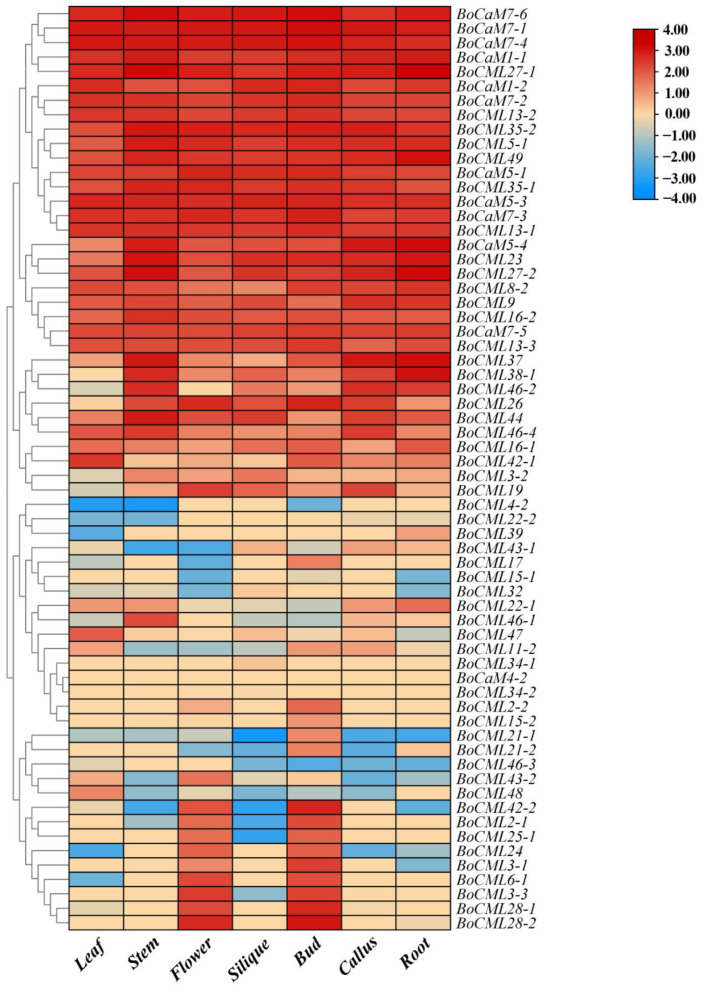
Differential expression patterns of *BoCaM*/*BoCML* genes in leaf, stem, flower, silique, bud, callus, and root of *B. oleracea*.

To determine the expression profiles of *BoCaM*/*BoCML* genes in leaves, stems, flowers, siliques, buds, calli, and roots, the RNA-Seq dataset (GSM1052958-964) was used for a comprehensive analysis across these tissues. The expression of 64 *BoCaM*/*BoCML* genes across different tissues was detected. The heatmap of the expression profiles for *BoCaM*/*BoCML* genes was generated using log2 FPKM values (Figure 5). The results show that most *BoCaM* genes (except *BoCaM4-2*) exhibited higher expression levels in all tissue compared to *BoCML* genes. *BoCaM4-2* was not detected in any tissues, suggesting that its expression may be specific to certain developmental stages. Specifically, *BoCML27-1*, *BoCML27-2*, *BoCML35-1*, *BoCML35-2*, and *BoCML49* exhibited relatively higher expression in all tissues compared to other *BoCML* genes, suggesting that their functions may be more extensive. *BoCML2-2* and *BoCML15-2* were expressed only in flowers and buds, suggesting that they may be involved in the development of floral organs or other related biological processes. *BoCML3-1*, *BoCML15-1*, and *BoCML28-2* were expressed in flowers, buds, and roots, implying that they play a vital role in the morphogenesis of *B. oleracea*. *BoCML3-3* and *BoCML25-1* were specifically expressed in flowers, buds, and siliques, indicating their essential roles in the reproductive regulation of *B. oleracea*. *BoCML34-1* and *BoCML34-2* were specifically expressed in siliques, suggesting that they play a unique role in silique development. Additionally, the expression of *BoCML39* was detected only in leaves and roots, highlighting its pivotal role in nutrient supply in *B. oleracea*.

### 2.6. The Relative Expression of BoCaM/BoCML Genes Under H. parasitica Infection

**Figure 6:**
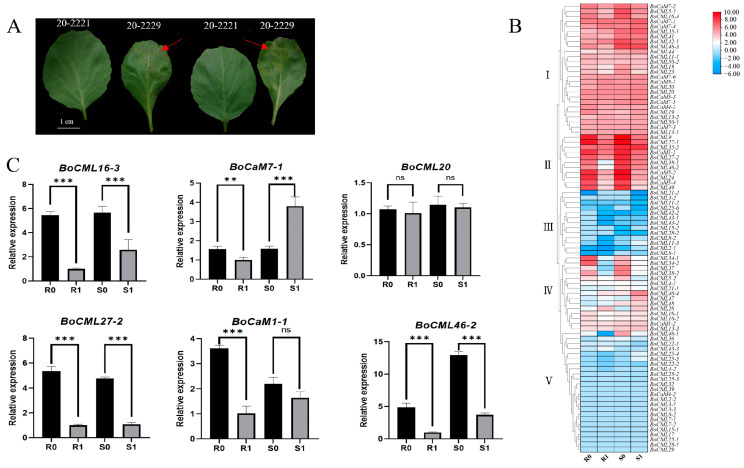
Phenotypes, heatmap, and RT-qPCR analysis related to the response of *BoCaM*/*BoCML* genes to *H. parasitica* infection. (**A**) Phenotypes of two cabbage cultivars before and four days after *H. parasitica* infection. The red arrows indicate the downy mildew-infected lesions (caused by *Peronosporaceae*) in the 20-2229 experimental material. (**B**) Heatmap of *BoCaM*/*BoCML* gene expression before and four days after *H. parasitica* infection. (**C**) qRT-PCR expression patterns of BoCaM/BoCML genes in *B. oleracea* before and four days after *H. parasitica* infection. Stars above the bars indicate significant differences among treatments. “ns” denotes no significant difference. Two stars (**) indicate a significant level (*p* < 0.01), and three stars (***) denote a highly significant level (*p* < 0.001).

To investigate the differential responses of *BoCaM*/*BoCML* genes in *B. oleracea* under *H. parasitica* infection, we conducted inoculation experiments using the resistant inbred line 20-2221 and the susceptible inbred line 20-2229. The symptoms of downy mildew infection in lines 20-2221 and 20-2229 were observed both before inoculation and four days post-inoculation. Four days after inoculation, no symptoms were observed on the abaxial leaf surface of 20-2221, whereas the abaxial leaf surface of 20-2229 exhibited not only lesions but also spore germination (Figure 6A). To investigate the role of *BoCaM* and *BoCML* genes in cabbage’s response to *H. parasitica* infection, RNA-seq was performed on resistant and susceptible leaves collected both before and four days after inoculation. Based on the RNA-seq data (BioProject ID: PRJNA1146208), the differential expression of *BoCaM* and *BoCML* genes in response to *H. parasitica* infection was analyzed. Using the differential expression data of *BoCaM* and *BoCML* genes in *B. oleracea* collected before and four days after *H. parasitica* infection, a heatmap of gene expression was generated (Figure 6B). The heatmap revealed that all *BoCaM*/*BoCML* genes clustered into five groups based on their responses to *H. parasitica* infection in both resistant and susceptible varieties. Genes in Cluster I (26) exhibited high expression levels in both resistant and susceptible materials but did not show changes in response to *H. parasitica* infection, suggesting that these genes are not involved in the defense response to *H. parasitica* infection. Genes in Cluster II (11) also displayed high expression in the leaves, but their expression significantly decreased in both resistant and susceptible materials four days post-infection, indicating that *H. parasitica* infection suppresses their expression. Genes in Clusters III (13) and IV (15) exhibited low expression levels in the leaves and similarly showed reduced expression in response to *H. parasitica* infection. Genes in Cluster V (24) were almost undetectable in the leaves, consistent with previously observed tissue-specific expression patterns. Six *BoCaM*/*BoCML* genes were randomly selected for qRT-PCR validation, and the results confirmed the reliability of the RNA-seq data (Figure 6C). Additionally, to identify the genes with the most significant response to downy mildew infection, RNA-seq and qRT-PCR results were combined, revealing that *BoCML46-2* exhibited the most significant reduction in expression following infection. Following infection by *H. parasitica*, *BoCML46-2* expression decreased nearly fivefold in both 20-2221 and 20-2229. Thus, it is hypothesized that *BoCML46-2* is a key *BoCaM*/*BoCML* gene that responds to *H. parasitica* infection.

## 3. Discussion

In this study, a genome-wide identification of *BoCaM* and *BoCML* genes in cabbage was conducted, resulting in the identification of 14 *BoCaM* and 75 *BoCML* genes, along with their collinearity, structures, chromosomal locations, and expression patterns across different tissues. Additionally, the differential expression profiles of *BoCaM* and *BoCML* genes in response to *H. parasitica* infection were analyzed. This study provides comprehensive insights into the *BoCaM* and *BoCML* gene families in cabbage.

The EF-hand motif is a conserved feature in both *CaM* and *CML* genes. Whereas *CaM* genes consistently contain four EF-hand motifs, the number of EF-hand motifs in *CML* genes varies from one to six [12,13]. The number of EF-hand motifs in *CML* genes varies across plant species. For instance, *CmCML13* and *AtCML43* contain three EF-hand motifs, whereas *MsCML10* and *CpCML15* possess four, which are involved in biotic and abiotic stress responses, respectively [24,25,26,27]. These imply that the variation in EF-hand motif numbers may play pivotal roles in differential stress response mechanisms. Interestingly, *BoCML* genes contain between two and four EF-hand motifs. Most paralogous genes contain the same number of EF-hand motifs (Figure 1); however, within the *BoCML46* family, *BoCML46-2* has three EF-hand motifs, whereas *BoCML46-1*, *BoCML46-3*, and *BoCML46-4* each have only two. This variation suggests that *BoCML46-2* may be involved in a broader range of pathways compared to the other three paralogous genes.

Genes that lack introns or have fewer introns are generally considered to be expressed more rapidly in plants, enabling a quicker response to biotic and abiotic stressors [28]. Most *BoCML* genes (52) have only one exon and lack introns, whereas *BoCaM* genes typically contain one to three introns. Additionally, most paralogous genes exhibit similar gene structures (e.g., *BoCML13-1* to *BoCML13-3* and *BoCML46-1* to *BoCML46-4*), although exceptions do exist. For example, *BoCML25-2* and *BoCML25-3* each contain one intron, whereas the other *BoCML25* paralogs lack introns. The majority of the *BoCML* genes identified in this study contain only one exon, and these genes may play a key role in the rapid response to various abiotic and biotic stresses.

Previous studies have demonstrated that *CaM*/*CML* genes play a crucial role in plant responses to pathogen infection [29]. Knockout of *OsCML31* has been shown to increase the susceptibility of rice to *Meloidogyne graminicola* [30]. As a positive regulatory factor, the overexpression of *AhCML69* in tobacco leaves enhances resistance to *Ralstonia solanacearum* infection and induces the expression of defense-related genes [31]. *CML43* serves as a key mediator of Ca^2+^-dependent signaling in the plant immune response to bacterial pathogens [24]. Overexpression of the rice gene *OsCaML2* confers resistance to *Xanthomonas oryzae* pv. *Oryzae* [21]. *AtCML46*, together with *AtCML47*, negatively regulates salicylic acid accumulation and immunity in *Arabidopsis.* The *cml46* and *cml47* double mutant displays increased resistance to *Pseudomonas syringae* and exhibits altered expression patterns of key immune regulators [32]. In this study, all *BoCaM*/*BoCML* genes responsive to *H. parasitica* infection were down-regulated, with *BoCML46-2* exhibiting a significant fivefold reduction in expression. Thus, it is hypothesized that *BoCML46-2* functions as a negative regulator in response to *H. parasitica* infection. The findings of this study will facilitate further investigation into the functions of *BoCaM*/*BoCML* genes in cabbage, particularly those related to responses to biotic and abiotic stressors.

## 4. Materials and Methods

### 4.1. Genome-Wide Identification of BoCaM/BoCML Genes in B. oleracea

The protein sequences of *AtCaM/AtCML* and *OsCaM/OsCML* genes were obtained from their respective databases based on previous research [15,33,34,35]. The BLASTP program (https://blast.ncbi.nlm.nih.gov/, accessed on 6 July 2024) was used to identify *BoCaM/BoCML* genes in *B. oleracea* genome by comparing them with the protein sequences of *AtCaM/AtCML* and *OsCaM/OsCML* genes [36,37]. The sequences of the 89 candidate *BoCaM*/*BoCML* genes were subjected to domain analysis using InterPro, the Conserved Domain Database, Pfam, and SMART, with the EF-hand domain IDs being cd00051, PF00036, IPR002048, and SM00054, respectively [38,39,40,41]. False *BoCaM*/*BoCML* gene candidates were filtered out based on conserved domains and the number and characteristics of EF-hand motifs, leading to the identification of 14 *BoCaM* genes and 75 *BoCML* genes. Based on the homologous sequences of these genes in *Arabidopsis*, the genes were designated sequentially as *BoCaM1-1* to *BoCML50-2*.

### 4.2. Prediction of Basic Infomation of BoCaM/BoCML Genes

To obtain information on the amino acid count, molecular weight (MW), theoretical isoelectric point (pI), instability index, aliphatic index, and grand average of hydropathicity (GRAVY) for the *BoCaM* and *BoCML* proteins, their sequences were submitted to ProtParam [42]. Each predicted *BoCaM*/*BoCML* genes was analyzed for EF-hand motifs using the InterPro tool (https://www.ebi.ac.uk/interpro/, accessed on 6 July 2024), and the GSDS 2.0 (Gene Structure Display Server; https://gsds.gao-lab.org/Gsds_help.php, accessed on 10 October 2024.) tool was used to analyze the exons and introns of the genes [39]. The PlantCARE analysis (https://bioinformatics.psb.ugent.be/webtools/plantcare/html/, accessed on 6 July 2024) was performed with default parameters, focusing on core promoter elements and stress-responsive motifs (Supplementary Table S4).The BUSCA (Bologna Unified subcellular Component Annotator) tool was used to predict the subcellular localization of each *BoCaM/BoCML* gene, with the taxonomic origin set to eukaryotic plants [43].

### 4.3. Phylogenetic Analysis

Phylogenetic analysis of *BoCaM/BoCML* genes was conducted using MEGA 7.0 software. Multiple sequence alignment was performed with ClustalW using default parameters, and a neighbor-joining phylogenetic tree was constructed based on p-distance with bootstrap values of 1000 replicates [44]. The protein sequences of *AtCaM*/*AtCML* genes in *Arabidopsis* and *OsCaM*/*OsCML* genes in *Oryza sativa* L. were obtained from the *Arabidopsis* Genome Database (The Arabidopsis Information Resource, TAIR; https://www.arabidopsis.org/, accessed on 6 July 2024) and the rice genome database (The Institute for Genomic Research, TIGR; http://rice.plantbiology.msu.edu/, accessed on 6 July 2024) according to precious research [15,34]. Phylogenetic trees for *BoCaM*/*BoCML* genes, *AtCaM*/*AtCML* genes, and *OsCaM*/*OsCML* genes were constructed using the same methods as described above.

### 4.4. Chromosome Location and Collinearity Analysis

The genome GFF3 file and the *BoCaM/BoCML* gene ID file, both downloaded from the *Brassica oleracea* genome JZS 2.0 (BRAD: http://brassicadb.cn/#/, accessed on 6 July 2024), were submitted to the functional module “Gene Location Visualize from GTF/GFF” in TBtools (v2.210) to map the chromosome localization of *BoCaM*/*BoCML* genes. The genome FASTA files for *B. oleracea* and *Arabidopsis* were submitted to the functional module “Fasta Stats” of TBtools to obtain the respective genome annotation files. Subsequently, the genome annotation files and FASTA files of *B. oleracea* and *Arabidopsis* were submitted to the functional module “One Step MCScanX-Super Fast” for collinearity analysis [45].

### 4.5. Expression Analysis of BoCaM/BoCML Genes Using RNA-Seq Data

To analyze the tissue-specific expression patterns of *BoCaM*/*BoCML* genes, RNA-seq data from various tissues, including leaves, stems, flowers, siliques, buds, calli, and roots, were downloaded from the NCBI database (GSM1052958-964). To analyze the expression patterns of *BoCaM*/*BoCML* genes in response to *H. parasitica* infection, leaves were collected from both resistant line 20-2221 and susceptible line 20-2229 at two time points: before inoculation and four days post-inoculation. Transcriptome sequencing was performed on these samples, and the RNA-seq data have been uploaded to NCBI (BioProject number: PRJNA1146208). Gene expression levels were calculated using FPKM values, and the FPKM algorithm was employed to normalize the gene expression data. Heat maps of hierarchical clustering were constructed using TBtools software [45].

### 4.6. Plant Materials and Treatments

*H.parasitica* used in this research was propagated and preserved by our laboratory for many years. Before inoculation, spores were harvested from the susceptible plant variety 20-2229, and an inoculum of 1 × 10^5^ spores/mL was prepared. The seeds of resistant variety 20-2221 and susceptible variety 20-2229 were sown in seedling trays, each measuring 10 cm by 10 cm, and filled with a sterilized substrate conducive to germination. *H. parasitica* inoculation was carried out using a sprayer when the seedlings had developed two true leaves. At this stage, the prepared spore suspension was evenly applied to the underside of the leaves. Each treatment included 30 seedlings, with 10 seedlings constituting one biological replicate. After inoculation, the plants were subjected to a 24 h dark incubation period. Subsequently, the plants were transferred to a greenhouse for routine cultivation under conditions of 16 h of light and 8 h of darkness at a temperature of 23–25 °C. Six days later, the plants underwent another 24 h dark incubation, with the relative humidity maintained at 95%.

### 4.7. Total RNA Extraction, cDNA Synthesis, and qRT-PCR Analysis

Total RNA was extracted from cabbage samples using the TIANGEN RNAprep Pure Plant Kit according to the supplier’s instructions (Transgen, Beijing, China). Then, the RNA purity and quality were assessed using a spectrophotometer (BioDrop, Cambridge, UK) and 1% formaldehyde gel electrophoresis. First-strand cDNA was synthesized using the FastKing RT Kit (TIANGEN, Beijing, China) following the manufacturer’s instructions. Specific primers for *BoCaM*/*BoCML* genes were designed using Premier 3.0 (Supplementary Table S3). qRT–PCR was performed using a TransStart Top Green qPCR SuperMix Kit (TransGen Biotech, Beijing, China) on a CFX96 Real-Time System (Bio-Rad, Hercules, CA, USA). Three technical and biological replicates were conducted for each reaction.

## 5. Conclusions

In this study, 14 *BoCaM* and 75 *BoCML* genes were identified in the *B. oleracea* genome and classified into six subgroups (Groups I–VI). Through bioinformatics and qRT-PCR analyses, the gene structures, phylogenetic relationships, chromosomal locations, gene duplications, and expression patterns of *BoCaM*/*BoCML* genes were examined. Eleven *BoCaM*/*BoCML* genes were down-regulated in response to *H. parasitica* infection, with *BoCML46-2* exhibiting the most significant decrease, nearly fivefold, in both resistant and susceptible varieties, suggesting that *BoCML46-2* may play a crucial role in the response to *H. parasitica*. This study provides comprehensive genome-wide information on *BoCaM*/*BoCML* genes in cabbage, which will facilitate the identification of their roles in cabbage growth, development, and stress responses.

## Data Availability

All data generated or analyzed during this study are included in this published article and its Supplementary Information Files. The raw sequencing data used during this study are available in the NCBI SRA database (BioProject number: PRJNA1146208). The *B. oleracea* reference genome ‘Braol JZS V2.0’ used in this study can be found at the link http://brassicadb.cn/#/, accessed on 10 July 2024. The *A. thaliana* genome can be found at the link https://www.arabidopsis.org/index.jsp, accessed on 6 July 2024. The protein database of National Center for Biotechnology Information (NCBI) can be found at the link https://www.ncbi.nlm.nih.gov/, accessed on 6 July 2024. All these databases are open to public access.

## Supplementary Materials

The following supporting information can be downloaded at: https://www.mdpi.com/article/10.3390/ijms26073208/s1.
